# The clinical application of shared decision-making in emergency surgery: a scoping review protocol

**DOI:** 10.1136/bmjopen-2025-104030

**Published:** 2025-09-17

**Authors:** Carly Nichola Bisset, Celia Keane, Tracey McKee, Rachel John-Charles, Cameron Iain Wells, Susan Joan Moug

**Affiliations:** 1 Dept of General Surgery, Golden Jubilee National Hospital, Clydebank, UK; 2 University of Glasgow, Glasgow, UK; 3 Dept of General Surgery, Royal Alexandra Hospital, Paisley, Scotland, UK; 4 Department of Surgery, The University of Auckland, Auckland, Auckland, New Zealand; 5 Te Whatu Ora - Te Tai Tokerau District, Whangarei, New Zealand; 6 NHS Greater Glasgow & Clyde Knowledge Services, Glasgow, Scotland, UK

**Keywords:** Emergency Service, Hospital, SURGERY, Clinical Decision-Making, Decision Making, Patient-Centered Care

## Abstract

**Abstract:**

**Introduction:**

Shared decision-making (SDM) between clinicians and patients is considered ‘best practice’. There is limited evidence regarding SDM in surgery, particularly in the emergency setting. Emergency SDM may be particularly challenging due to: time pressures, the patient’s underlying condition and the nature of the patient-surgeon interaction. However, emergency surgery arguably has a greater need for SDM due to the likelihood of disparate outcomes from intervention, which is dependent on the various treatment options available. This is necessary for patients to make informed decisions regarding their treatment of surgical pathology. The primary objective of this scoping review is to understand the extent and type of evidence in relation to SDM in emergency surgery to determine methods for improving SDM.

**Methods:**

Any studies reporting SDM in emergency surgery on adult patients (age >18 years) will be included. EMBASE, Medline, Cochrane, CINAHL and Scopus databases will be searched for articles with no language or date limits. Studies will be screened by two independent reviewers, with consensus met prior to data extraction. Data extracted to include study design, details of study population, tools used to measure SDM, prevalence of SDM and barriers and enablers for SDM.

A systematic narrative synthesis will be performed following JBI (Joanna Briggs Institute) guidance. These will summarise findings of included studies. The findings may inform future research into facilitating implementation of SDM in emergency surgery.

**Ethics and dissemination:**

This study does not require ethical approval. Final findings will be submitted for peer-reviewed publication and presentation at surgical conferences.

**STRENGTHS AND LIMITATIONS OF THIS STUDY**

- This scoping review will allow the inclusion of data from qualitative sources which can provide a more comprehensive understanding of the challenges of implementing shared decision-making (SDM) in emergency surgery.
- By including all surgical specialties, lessons may be gathered from a variety of patients and different risk perceptions; however, this may limit the generalisability of results to specific instances (eg, SDM may differ due to risk of specific post-operative morbidity or mortality).
- A further strength of this protocol was close adherence to JBI scoping review guidelines.

## Introduction

 Shared decision-making (SDM) has been defined as the ‘process in which clinicians and patients work together to clarify treatment, management or self-management support goals, sharing information about options and preferred outcomes with the aim of reaching mutual agreement on the best course of action’.^1^ There are numerous benefits of engaging in SDM. For example, SDM is associated with improved patient satisfaction, resulting in increased compliance with treatment.^2^ Through discussion of the anticipated benefits of treatment options, risks of treatment options and exploration of alternatives (including doing nothing), patient autonomy is upheld. The process of SDM includes three key aspects. First, the provision of reliable and balanced evidence-based information which outlines treatment options, outcomes and uncertainties. Second, the provision of decisional support with a clinician to clarify options and preferences. Finally, the provision of a system to record, communicate and implement the patient’s preferences.^1^ While SDM is considered best practice (ie, the ‘gold standard’), research into SDM practices in medicine is limited and fraught with bias,^3^ and the practical delivery of SDM in clinical settings can be challenging.^4^ Attempts to quantify SDM in surgical contexts report variable results. A 2018 systematic review of 32 studies reported that 36% of participants perceived there to have been SDM in consultations where elective surgery was a treatment option. However, there was a disparity between surgeon and patient perception, with surgeons more frequently reporting decisions to be shared (43.6% of surgeons vs 29.3% of patients), rather than patient- or surgeon-driven.^5^ This raises questions regarding what SDM means to different stakeholders and how best to accurately measure SDM in clinical practice.

SDM is particularly challenging in the emergency setting due to time pressures, the acuity of the patient’s clinical condition, greater uncertainty around post-operative outcomes and the lack of guidance on how best to quantify risk in high-risk surgical patients in terms which are best understood by patients.^6^ Pain, distress, delirium or medication usage, as well as socioeconomic status and language barriers, are likely to influence conversations around treatment options and risk and may influence decision-making abilities.^4 7 8^ This is in stark contrast to the planned, elective surgical setting where shared decisions may be made as part of an ongoing process over an extended period of time within an established therapeutic relationship.^9^,^11^ Despite these challenges, SDM is of great importance in the emergency setting,^12^ where patients and/or their families are required to make urgent and often ‘high stakes’ decisions, including decisions around treatment escalation plans and ‘ceilings of care’.^13^ Exploring SDM in the emergency setting has recently been highlighted as a peer-reviewed research priority in emergency general surgery (‘What influences decision-making for emergency surgery for the surgeon, patient and family?’).^14^ The 2018 systematic review included a single retrospective assessment of SDM in an emergency surgery involving a decision between reconstruction vs amputation after an open tibial fracture.^15^ Furthermore, recent scoping reviews of SDM across all surgical specialties found mixed quality of studies with none evaluating decision-making preferences in emergency surgery.^16 17^ Therefore, it was considered necessary to conduct a further contemporaneous and more comprehensive scoping review paying specific attention to SDM in emergency surgery. A scoping review was considered more appropriate than a systematic review, as there is still emerging evidence regarding approaches in improving SDM which are currently not suitable for systematic review. Furthermore, we aim to examine how research is conducted in shared decision-making in surgery, a process which is more suited to a scoping review.^18^

Therefore, the primary aim of this scoping review is to assess the extent and type of evidence regarding the use of SDM in the emergency surgical setting. Secondary aims include identification of barriers to engaging in SDM and identification of strategies for improving SDM in emergency surgical settings.

## Review question(s)

1. What evidence is available regarding SDM in emergency surgery?
2. What is the prevalence of SDM in emergency surgery?
3. What are the barriers to SDM in emergency surgery?
4. What are potential strategies for improving SDM in emergency surgery settings?

## Methods and analysis

The protocol was developed according to JBI guidance for scoping reviews.^18^ The proposed scoping review will also be conducted in accordance with JBI methodology.^18^ Data collection for this scoping review is underway. The protocol is registered on the Open Science Framework (DOI 10.17605/OSF.IO/QUXEK). The Preferred Reporting Items for Systematic reviews and Meta-Analyses extension for Scoping Reviews (PRISMA-ScR) checklist will be used to report the findings of this scoping review.

### Concept

For the purposes of this review, SDM will be defined as the ‘process in which clinicians and patients work together to clarify treatment, management or self-management support goals, sharing information about options and preferred outcomes with the aim of reaching mutual agreement on the best course of action’.^1^

### Eligibility criteria

Studies reporting assessment of SDM in adults >/= 18 years old in emergency surgery settings (all specialties) will be included in this scoping review.

Mixed study populations including both patients undergoing elective and emergency surgery will be considered for inclusion if it is possible to differentiate the outcomes for elective and emergency settings. This review will use the population, concept and context (PCC) framework suggested by JBI. This is described in table 1. The included study population will be patients only as per the definition of SDM,^1^ with family members or caregivers excluded from primary outcomes analysis. Mixed study populations including patients and their relatives/carers will be considered for conclusion if it is possible to differentiate outcomes between the two groups as this may address our secondary aims.

**Table 1:** PCC Framework for Shared Decision-Making in Emergency Surgery Scoping Review

*Population*	*Adults >/= 18 years old undergoing emergency surgery (all specialties*)
*Concept*	*Use of shared decision making*
*Context*	*Health research*

PCC, population, concept and context.

### Exclusion criteria

The exclusion criteria of this study is as follows:

- Reviews, letters to editor, editorials, suggested models of care, patient education handouts, decision-making tools, paediatric literature, animal studies.
- Studies assessing SDM in elective surgical settings only
- Studies assessing SDM in medical rather than surgical settings.
- Studies assessing SDM in paediatric populations (patient age <18 years).

### Types of sources

This scoping review will consider randomised and non-randomised study designs including prospective and retrospective cohort studies, case-control studies, case series and cross-sectional studies. Qualitative studies that investigate decision-making in emergency surgical settings will also be considered for inclusion. Animal studies will be excluded.

### Search strategy

The search strategy will aim to locate published and unpublished studies. The search strategy was developed based on the strategy used in the prior scoping review^16^ with additional guidance from a medical librarian (online supplemental appendix I). The search strategy, including all identified keywords and index terms, will be adapted for each included database and/or information source. The reference lists of all included sources of evidence will be screened for additional studies.

Studies reported in any language will be included. There will be no date limit applied to the searches. The databases to be searched include OVID Embase, OVID MEDLINE, EBSCOhost CINAHL, The Cochrane Library incorporating the Cochrane Database of Systematic Reviews and the Cochrane Central Register of Controlled Trials, and Scopus. Study authors will be contacted if required to obtain further information. Unpublished studies will be sourced via a review of conference abstracts found during the database searches, and a general internet search using keywords.

### Study/source of evidence selection

Following the search, all identified citations will be collated and uploaded into the Systematic Review Accelerator for deduplication.^19^ The Rayyan research tool will be used for the screening process, which is an online ‘software as a service’ application.^20^ Following a pilot test, titles and abstracts will then be screened by two independent reviewers (two of CB, CW, RJC) for assessment against the inclusion criteria for the review. Potentially relevant sources will be retrieved in full and their citation details imported into Rayyan. The full text of selected citations will be assessed in detail against the inclusion criteria by two independent reviewers (two of CB, RJC, CW). Reasons for exclusion of sources of evidence at full text that do not meet the inclusion criteria will be recorded and reported in the scoping review. Any disagreements that arise between the reviewers at each stage of the selection process will be resolved through discussion, or with an additional reviewer by (CK). The results of the search and the study inclusion process will be reported in full in the final scoping review and presented in a PRISMA-ScR flow diagram.^21^

### Data extraction

Data will be extracted from papers included in the scoping review by two independent reviewers (CB, RJC, CW) using a data extraction tool developed by the team of reviewers. The PCC framework for data extraction will be followed to construct clear criteria for the scoping review.^19^ The data extracted will include specific details about the study design, with data items including interventions studied and their application in emergency surgery. This may include the prevalence of SDM, barriers to implementation and current strategies for SDM in emergency surgery.

A draft extraction form is provided (online supplemental appendix II). The draft data extraction tool will be modified and revised as necessary during the process of extracting data from each included evidence source. Modifications will be detailed in the scoping review. Any disagreements that arise between the reviewers will be resolved through discussion or with an additional reviewer (CK). If appropriate, authors of papers will be contacted to request missing or additional data, where required.

Critical appraisal of individual sources of evidence will not be done as this is not required for scoping reviews. The anticipated start date for screening is August 2025.

### Data analysis and presentation

A narrative summary will accompany the tabulated results regarding potential barriers and enablers/strategies for SDM. If there is more than one study included which reports prevalence of SDM in emergency surgery, a pooled prevalence will be reported. The findings of this work will be used to guide a patient and public involvement exercise with the purpose of exploring SDM in the emergency setting.

### Patient and public involvement (PPI)

PPI representation was present within the design, conduct, reporting and dissemination plans of this scoping review protocol and the intended scoping review, with the aim of developing a further research study on improving SDM in emergency surgery.

## Ethics and dissemination

The results from the scoping review will be submitted for peer-reviewed journal publication and presentation at surgical conferences. The study findings will contribute to the development of future research by this study group on SDM in emergency surgery. The anticipated completion date of this study is February 2026.

## Discussion

The aim of this scoping review is to assess the quality and type of evidence surrounding SDM in emergency surgery. This is necessary to identify current barriers in its implementation and to identify potential strategies for its improvement. The scoping review may also identify what may work for specific specialties compared to others, which may allow more personalised approaches based on risk perception and patient preferences.

The Montgomery ruling in the UK, where patients with full mental capacity must legally be advised about all treatment options and their associated risks, in order for patients to make informed decisions, has led to increased emphasis on the importance of improving the quality of research in SDM. Therefore, the insights gained from this scoping review will contribute to improving our current understanding of SDM in emergent surgery and may inform the development of future research in SDM.

## Supplementary material

10.1136/bmjopen-2025-104030 online supplemental file 1

10.1136/bmjopen-2025-104030 online supplemental file 2

## References

[1] Coulter A, Collins A (2011). Making Shared Decision-Making a Reality.

[2] Thibau IJ, Loiselle AR, Latour E (2022). Past, present, and future shared decision-making behavior among patients with eczema and caregivers. JAMA Dermatol.

[3] Ubbink DT (2022). Shared decision-making should be a standard part of surgical care. Br J Surg.

[4] Ankuda CK, Block SD, Cooper Z (2014). Measuring critical deficits in shared decision making before elective surgery. Patient Educ Couns.

[5] de Mik SML, Stubenrouch FE, Balm R (2018). Systematic review of shared decision-making in surgery. Br J Surg.

[6] Stephens TJ, Pearse RM (2022). The limitations of shared decision-making in surgery. Br J Surg.

[7] Law J, Welch C, Javanmard-Emamghissi H (2020). Decision-making for older patients undergoing emergency laparotomy: defining patient and clinician values and priorities. Colorectal Dis.

[8] Kalsi D, Ward J, Lee R (2019). Shared decision-making across the specialties: much potential but many challenges. J Eval Clin Pract.

[9] Boss EF, Mehta N, Nagarajan N (2016). Shared decision making and choice for elective surgical care. Otolaryngol--head neck surg.

[10] Cooper Z, Courtwright A, Karlage A (2014). Pitfalls in communication that lead to nonbeneficial emergency surgery in elderly patients with serious illness: description of the problem and elements of a solution. Ann Surg.

[11] Bisset CN, Dames N, Oliphant R (2020). Exploring shared surgical decision-making from the patient’s perspective: is the personality of the surgeon important?. Colorectal Dis.

[12] Hess EP, Grudzen CR, Thomson R (2015). Shared decision-making in the emergency department: respecting patient autonomy when seconds count. Acad Emerg Med.

[13] Fried TR, Bradley EH (2003). What matters to seriously ill older persons making end-of-life treatment decisions?: a qualitative study. J Palliat Med.

[14] Vaughan EM, Pearson R, Wohlgemut JM (2022). Research priorities in emergency general surgery (EGS): a modified Delphi approach. World J Emerg Surg.

[15] Aravind M, Shauver MJ, Chung KC (2010). A qualitative analysis of the decision-making process for patients with severe lower leg trauma. Plast Reconstr Surg.

[16] Shinkunas LA, Klipowicz CJ, Carlisle EM (2020). Shared decision making in surgery: a scoping review of patient and surgeon preferences. BMC Med Inform Decis Mak.

[17] Niburski K, Guadagno E, Mohtashami S (2020). Shared decision making in surgery: a scoping review of the literature. Health Expect.

[18] Peters MDJ, Godfrey C, McInerney P, Aromataris E, Munn Z (2024). JBI Manual for Evidence Synthesis.

[19] Clark J, Glasziou P, Del Mar C (2020). A full systematic review was completed in 2 weeks using automation tools: a case study. J Clin Epidemiol.

[20] Ouzzani M, Hammady H, Fedorowicz Z (2016). Rayyan-a web and mobile app for systematic reviews. Syst Rev.

[21] Tricco AC, Lillie E, Zarin W (2018). PRISMA Extension for Scoping Reviews (PRISMA-ScR): checklist and explanation. Ann Intern Med.

