# Supplementary material for: The clinical application of shared decision-making in emergency surgery: a scoping review protocol
Source: BMJ Open. 2025 Sep 17;15(9):e104030. doi: 10.1136/bmjopen-2025-104030 (PMC12458821; doi:10.1136/bmjopen-2025-104030)
Supplement: online supplemental file 1 [file bmjopen-15-9-s001.docx]

**Appendix I: Medline search strategy**

Ovid MEDLINE(R) ALL <1946 to July 11, 2025>

1      Decision Making/    110471

2      Decision Making, Shared/   2881

3      decision support techniques/  23679

4      Negotiating/ 7489

5      consensus/  25604

6      uncertainty/ 20217

7      Choice Behavior/    36715

8      "Dissent and Disputes"/      6201

9      Patient Preference/ 12148

10    Patient Participation/   31240

11    Patient Satisfaction/ 95497

12    patient-centered care/ or patient navigation/  26244

13    (decision* adj3 (conflict* or regret* or support* or preparedness or preparation or assistance or needs or make* or making* or made or tool* or aid or aide or aids or shared or sharing or resource* or informed or directed or paternalistic or cooperative or collaborative or consensus or involved or involvement or uncertainty or participation or (support* adj3 technique*))).ti,ab,kf.      362473

14    choice behavio?r*1.ti,ab,kf. 2316

15    (patient preference*1 or patient choice*1).ti,ab,kf. 16671

16    (patient* adj3 (education* or engage* or participat* or communicat* or empower* or activation* or involvement* or counsel* or advice* or guidance* or advise* or facilitat* or barrier* or ((center* or centre*) adj care*))).ti,ab,kf.       239850

17    1 or 2 or 3 or 4 or 5 or 6 or 7 or 8 or 9 or 10 or 11 or 12 or 13 or 14 or 15 or 16     850694

18    exp Specialties, Surgical/   229417

19    Emergencies/  44364

20    18 and 19    1095

21    ((emergency or emergencies or emergent or urgent or expedite*) adj3 (surger* or surgical* or operation or operations or operative or procedure or procedures or reoperat* or laparoscop* or laparotom* or resection*)).ti,ab,kf.  55792

22    20 or 21       56525

23    17 and 22    2560

24    Animals/      7699996

25    Humans/      22838578

26    24 and 25    2377707

27    24 not 26     5322289

28    23 not 27     2553
