# Supplementary material for: The clinical application of shared decision-making in emergency surgery: a scoping review protocol
Source: BMJ Open. 2025 Sep 17;15(9):e104030. doi: 10.1136/bmjopen-2025-104030 (PMC12458821; doi:10.1136/bmjopen-2025-104030)
Supplement: online supplemental file 2 [file bmjopen-15-9-s002.docx]

**Appendix II: Data extraction instrument**

Only append the JBI or non-JBI data extraction instrument if the standardized tool has been modified in any way, otherwise simply cite the tool used in the text. Any modifications made to the instrument should also be described in the text.

| **Citation Details** | **Year** | **Country of Origin** | **Aims/Purpose** | **Population & Sample Size** | **Context  (e.g. speciality, emergency vs mixed population)** | **Outcomes** | **Key Findings** |
| --- | --- | --- | --- | --- | --- | --- | --- |
| Author  PubMED |  |  |  |  |  | Outcome used to measure SDM  Reference for SDM  Prevalence of SDM | e.g. barriers, method of improving shared decision-making, other conclusions |
|  |  |  |  |  |  |  |  |
|  |  |  |  |  |  |  |  |
|  |  |  |  |  |  |  |  |
|  |  |  |  |  |  |  |  |
|  |  |  |  |  |  |  |  |
