# Supplementary material for: The clinical application of shared decision-making in emergency surgery: a scoping review protocol
Source: BMJ Open. 2025 Sep 17;15(9):e104030. doi: 10.1136/bmjopen-2025-104030 (PMC12458821; doi:10.1136/bmjopen-2025-104030)
Supplement: Supplementary file 3 [file bmjopen-2025-104030.reviewer_comments.pdf]

PEER REVIEW HISTORY

BMJ Open publishes all reviews undertaken for accepted manuscripts. Reviewers are asked to complete a checklist review form and are provided with free text boxes to elaborate on their assessment. These free text comments are reproduced below.

ARTICLE DETAILS

Title (Provisional)

The clinical application of shared decision-making in emergency surgery: a scoping review protocol

Authors

Bisset, Carly Nichola; Keane, Celia; McKee, Tracey; John-Charles, Rachel; Wells, Cameron Iain; Moug, Susan Joan

---

VERSION 1 - REVIEW

**Reviewer**	**1**
**Name**	**Simons, Mary**
**Affiliation**	**Macquarie University, Library**
**Date**	**13-May-2025**
**COI**	**None**

This is an important topic that is well suited to a scoping review. The following comments are suggestions for improvements to the manuscript in keeping with current guidelines:

Introduction:

Include more background and a rationale about why SDM is considered good practice;

Also include a comment about situations where you do or don't use SDM in emergency surgery, for example, is its use preferred only when there is equipoise or are there other factors to consider?

The JBI Guideline suggests that the PRISMA ScR checklist is used, not just the flow diagram as you have indicated. (p11 line 43)

The PCC (Population, Concept, Context) mentioned in your data extraction section (p.11 line 58) forms the framework for the search and should be included earlier in the inclusion criteria in accordance with the JBI scoping review protocol guideline (ie. "The introduction should conclude with an overarching review objective that captures and aligns with the core elements/mnemonic of the inclusion criteria (e.g. PCC)". The PRISMA ScR checklist includes it in the Introduction as the objectives are outlined – see Item 4 of the Checklist.

Methods

Should the "family" or "caregiver(s)" be included as part of the population and inclusion criteria? "Families" are only mentioned once in your manuscript, (page 7 line 19) and "caregiver" is not mentioned at all. Yet they would have an important role in any emergency surgery shared decision-making situation, especially if the patient is not able to engage with the process. Consider including them in your search strategy.

Search Strategy– Some search terms are too broad. For example, the MeSH term 'Decision making/' will retrieve non-SDM studies. But 'Specialties, surgical/' should be exploded (exp Specialties, surgical/) to retrieve important specialties such as neurosurgery and thoracic surgery etc. You have stated you are including all specialties. You may wish to consult a librarian who can help you to review your search strategy.

The search steps taken to remove animal studies should be described in the section on the search strategy as it forms part of the exclusion criteria. Not sure if you need it though as it only excluded 7 studies that could be easily screened out.

Formatting issues

Reference list – journal names are not consistently formatted with capital letters

---

VERSION 1 - AUTHOR RESPONSE

Reviewer: 1
Ms. Mary Simons, Macquarie University

Comments to the Author:
This is an important topic that is well suited to a scoping review. The following comments are suggestions for improvements to the manuscript in keeping with current guidelines:

Introduction:
3. ☐ Include more background and a rationale about why SDM is considered good practice;
The benefits of SDM are now included within the introduction with a new reference included.

4. ☐ Also include a comment about situations where you do or don't use SDM in emergency surgery, for example, is its use preferred only when there is equipoise or are there other factors to consider?
Thank you. The authors felt that the description within paragraph 2 of the introduction explored the main issues surrounding SDM in emergency settings as such has not been expanded upon further.

5. ☐ The JBI Guideline suggests that the PRISMA ScR checklist is used, not just the flow diagram as you have indicated. (p11 line 43)

Thank you for highlighting this. This has now been included as Appendix 3.

6. ☐ The PCC (Population, Concept, Context) mentioned in your data extraction section (p.11 line 58) forms the framework for the search and should be included earlier in the inclusion criteria in accordance with the JBI scoping review protocol guideline (ie. “The introduction should conclude with an overarching review objective that captures and aligns with the core elements/mnemonic of the inclusion criteria (e.g. PCC)”. The PRISMA ScR checklist includes it in the Introduction as the objectives are outlined – see Item 4 of the Checklist.

This has now been included within the methods section and is summarised in Table 1.

Methods

7. ☐ Should the “family” or “caregiver(s)” be included as part of the population and inclusion criteria? “Families” are only mentioned once in your manuscript, (page 7 line 19) and “caregiver” is not mentioned at all. Yet they would have an important role in any emergency surgery shared decision-making situation, especially if the patient is not able to engage with the process. Consider including them in your search strategy.

The authors agree that the support networks of patients are important influencing factors in shared decision-making. However, the focus of shared decision-making is the relationship between the patient and the clinician (see definition of shared decision-making referenced in introduction). The influence of relatives / caregivers would be considered one of our secondary aims (“Secondary aims include identification of barriers to engaging in SDM”. The authors anticipate this will be elaborated on in further detail within the discussion section of the full scoping review manuscript, once data analysis has occurred.

Search Strategy

8. ☐ Some search terms are too broad. For example, the MeSH term ‘Decision making/’ will retrieve non-SDM studies. But ‘Specialties, surgical/’ should be exploded (exp Specialties, surgical/) to retrieve important specialties such as neurosurgery and thoracic surgery etc. You have stated you are including all specialties. You may wish to consult a librarian who can help you to review your search strategy.

Thank you for your suggestions. TM is a specialist librarian and was involved in our search strategy and is listed as a co-author. The broad subject headings and keywords around the concept of “Shared Decision-Making” have been retained to minimise the risk of missing relevant articles on a topic that can be described in many ways. The subject heading Specialties, surgical/ has now been exploded. Please see updated Search Strategy in Appendix 1, page 19.

9. ☐ The search steps taken to remove animal studies should be described in the section on the search strategy as it forms part of the exclusion criteria. Not sure if you need it though as it only excluded 7 studies that could be easily screened out.

Animal studies have been added under exclusion criteria at line 4, page 10.

Formatting issues

10. ☐ Reference list – journal names are not consistently formatted with capital letters

This has now been addressed. An additional reference has been inserted for the PRISMA SCR Checklist:

Tricco, AC, Lillie, E, Zarin, W, O'Brien, KK, Colquhoun, H, Levac, D, Moher, D, Peters, MD, Horsley, T, Weeks, L, Hempel, S et al. PRISMA extension for scoping reviews (PRISMA-ScR): checklist and explanation. Ann Intern Med. 2018,169(7):467-473.

VERSION 2 - REVIEW

**Reviewer**	**1**
**Name**	**Simons, Mary**
**Affiliation**	**Macquarie University, Library**
**Date**	**29-Jul-2025**
**COI**	

Your search appears rather dated as it was done in 2022. Search strategy: The MeSH term for surgical specialties has not been exploded as you indicated – line 18.

If you are not going to include studies involving family members or caregivers consider adding this group to the exclusion criteria. Would you include studies that included both patients and family members/caregivers?

Other minor comments:

Some minor editing suggestions. Include a page number for the section you have mentioned for reference 14: "What influences decision-making for emergency surgery for the surgeon, patient and family?"

You have Appendix II and Appendix 3. Use one consistent form of numbering.

Reference List: Some journal names are abbreviated and some are not. Please use the style recommended by this journal.

VERSION 2 - AUTHOR RESPONSE

Thank you for your comments, these have now all been addressed but are summarised below.

Strengths and Limitations have been updated to incorporate 1 sentence bullet points.

We have now incorporated an anticipated start date (August 2025) and completion date (February 2026) for the Methods section.

The search strategy was updated – this was a typo and has since been edited to reflect the most recent search. Thank you for highlighting this.

The search strategy has been updated to reflect the MeSH terms as indicated – this is found in Appendix I.

The inclusion and exclusion criteria has been updated to reflect the suggestions made –

“The included study population will be patients only as per the definition of shared decision-making [1], with family members or caregivers excluded from primary outcomes analysis. Mixed study populations including patients and their relatives/carers will be considered for conclusion if it is possible to differentiate outcomes between the two groups as this may address our secondary aims.”

The appendices now have consistent numbering as suggested. References have been updated.
